# Short and long term retention in antiretroviral care in health facilities in rural Malawi and Zimbabwe

**DOI:** 10.1186/1472-6963-12-444

**Published:** 2012-12-05

**Authors:** Freya Rasschaert, Olivier Koole, Rony Zachariah, Lut Lynen, Marcel Manzi, Wim Van Damme

**Affiliations:** 1 Institute of Tropical Medicine, Nationale straat 155, Antwerpen 2000, Belgium; 2 Médecins Sans Frontières, Operational Centre Brussels, Brussels, Belgium

**Keywords:** Retention, ART, HIV, SSA, Resource-limited settings, Short and long term

## Abstract

**Background:**

Despite the successful scale-up of ART services over the past years, long term retention in ART care remains a major challenge, especially in high HIV prevalence and resource-limited settings. This study analysed the short (<12 months) and long (>12 months) term retention on ART in two ART programmes in Malawi (Thyolo district) and Zimbabwe (Buhera district).

**Methods:**

Retention rates at six-month intervals are reported separately among (1) patients since ART initiation and (2) patients who had been on ART for at least 12 months, according to the site of ART initiation and follow-up, using the Kaplan Meier method. ‘Retention’ was defined as being alive on ART or transferred out, while ‘attrition’ was defined as dead, lost to follow-up or stopped ART.

**Results:**

In Thyolo and Buhera, a total of 12,004 and 9,721 patients respectively were included in the analysis. The overall retention among the patients since ART initiation was 84%, 80% and 77% in Thyolo and 88%, 84% and 82% in Buhera at 6, 12 and 18 months, respectively. In both programmes the largest drop in ART retention was found during the initial 12 months on ART, mainly related to a high mortality rate in the health centres in Thyolo and a high loss to follow-up rate in the hospital in Buhera. Among the patients who had been on ART for at least 12 months, the retention rates leveled out, with 97%, 95% and 94% in both Thyolo and Buhera, at 18, 24 and 30 months respectively. Loss to follow-up was identified as the main contributor to attrition after 12 months on treatment in both programmes.

**Conclusions:**

To better understand the reasons of attrition and adapt the ART delivery care models accordingly, it is advisable to analyse short and long term retention separately, in order to adapt intervention strategies accordingly. During the initial months on ART more medical follow-up, especially for symptomatic patients, is required to reduce mortality. Once stable on ART, however, the ART care delivery should focus on regular drug refill and adherence support to reduce loss to follow up. Hence, innovative life-long retention strategies, including use of new communication technologies, community based interventions and drug refill outside the health facilities are required.

## Background

Access to antiretroviral therapy (ART) continues to expand rapidly worldwide, especially in Sub-Saharan Africa (SSA) with a 30-fold increase in ART coverage since the end of 2003 [1]. This rapid ART scale-up process was facilitated through decentralisation of ART services, task shifting and involvement of the community in care delivery [2-5]. For those fortunate enough to receive ART, HIV/AIDS became a chronic life-long disease. Long term retention in ART care remains however a major challenge, particularly in SSA countries with limited resources and a severe shortage of health staff [2].

Two successive systematic reviews on retention in ART care showed a positive trend in overall retention rates over the past three years [6,7]. The first analysis (2007) reported a 62% retention rate at 24 months of treatment, in the later analysis (2010) this rate increased to 76%, probably due to the increased experience in handling large ART cohorts and a better tracing system for patients missing from care. Both analyses found a lower retention rate in patients recently initiated on ART (≤12m), mainly due to a high early mortality rate, whereas in the older cohorts the main reason for attrition was patients defaulting treatment [8,9]. Therefore it might be advisable to separate cohort analyses in short (≤12m) and long term (>12m) retention in care as they may require different types of interventions.

This study analysed the short and long term retention among the patients started on ART in two ART programmes supported by Médecins Sans Frontières (MSF) in Malawi, Thyolo district, and Zimbabwe, Buhera district, by separating the patients since ART initiation (including patients ≤12 months on ART) from the patients who had been on ART for at least 12 months.

## Methods

### Study setting and population

MSF started HIV projects in close collaboration with the Ministry of Health in Thyolo, one of the poorest districts in Southern Malawi, in 1997 and in Buhera, a poor rural district in the South-east of Zimbabwe, in 2004. Thyolo district has a population of 587,455 habitants, with an HIV prevalence of 14.0%, and 82,250 people living with HIV/AIDS (PLWHA) of whom 16,450 are estimated in urgent need of ART. Buhera district, with a population of 223,382 inhabitants, has an HIV prevalence of 19.7%, and 44,000 PLWHA of whom 8,800 are estimated in urgent need of ART, according to the WHO ART eligibility criteria – 2006 (Table 1).

**Table 1:** **Malawi and Zimbabwe**: **Population and HIV**-**related indicators**, **2009**

	**Malawi**[10]	**Thyolo**[10]	**Zimbabwe**[11]	**Buhera**[11]
Total population	15 million	587455	13.4 million	223382
HIV prevalence (15–45 years-old)	11.9%	14.0%	14.3%	19.7%
Total people living with HIV/AIDS	1785000	82250	1916200	44000
People in need of ART – CD4<200 cells/mm^3^	305805	16450	389895	8800
Active and alive on ART	198864	15016	218589	9721*
Year Start HIV programme		1997		2004
Year ART implementation		2003		2005
Year Decentralisation ART activities		2006		2006

* Buhera district attracts a large proportion of PLWHA from neighbouring districts.

**Figure 1:**
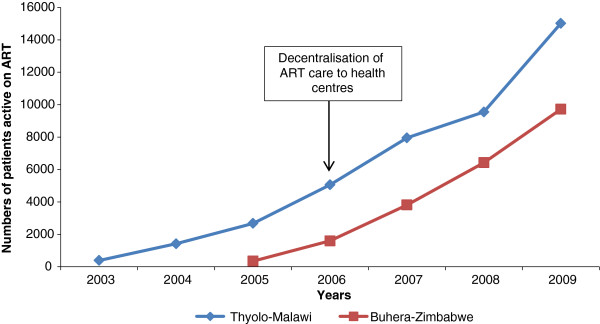
Total number of patients, active on ART per year – Thyolo–Malawi, 2003–2009 and Buhera–Zimbabwe, 2005–2009.

In both settings the health systems are weak, facing severe human resources for health shortages and, infrastructure and drug supply problems. In Thyolo and Buhera, ART was initially introduced at the district hospitals in 2003 and 2005, respectively. In 2006, ART care activities were decentralised to the peripheral health centres in both programmes to enable ART scale-up. This process implied shifting towards a public health approach with integration of ART services into the health centres, shifting of tasks to lower health cadres and increased community involvement. In both settings, the decentralisation process resulted in a steep increase in patients accessing ART. By December 2009, the programmes in Thyolo and Buhera counted respectively 15,016 and 9,721 patients on treatment (Figure 1). Both programmes involved community health care workers to trace patients who did not show up at their scheduled appointment.

All HIV infected patients, aged 15 years or older at the time of ART initiation were included in the analysis. They were divided into two or three groups according to the place of ART initiation and follow-up: (1) patients initiated and followed up in the hospital, (2) patients initiated and followed up in the health centres and (3) patients initiated in the hospital and later referred to the health centres. In Thyolo, patients referred from the hospital to the health centres were classified as transferred out. They could not be retrieved, however, as the datasets of the different sites could not be merged.

The study period of the two programmes differs depending on the data available. For both settings, the patients were included from the start of the ART availability (2003, Thyolo and 2005, Buhera) till September 2008 for Thyolo and December 2009 for Buhera.

### Data collection

In the district hospital of Thyolo, all the patients registered in the programme and initiated on ART are monitored through an electronic database, FUCHIA (Follow Up and Care of HIV infection and AIDS, Epicentre-MSF). In the decentralised ART sites, standardised and simplified monitoring tools – patients’ registers, master cards and individual identity cards – were used to monitor the ART outcomes [12].

In Buhera, a parallel monitoring system has been maintained over the past years. A paper-based system was used with several registers plus an electronic database system (FUCHIA), where patients in the decentralised ART sites were reported on a six-monthly basis.

### Data analysis

In both programmes, treatment outcomes are defined as: (1) ‘alive and on ART’ – patients initiated on ART and still followed up in one of the health facilities, (2) ‘died’ – patients who have died for any reason while on ART, (3) ‘lost to follow-up’ – patients on ART who have not attended the clinic for three months since the last scheduled appointment date, according to the national guidelines in both countries [13], (4) ‘stopped’ – patients who have stopped ART for any reason, (5) ‘transferred out’ – patients who have been transferred permanently to another health facility. For this analysis, ‘retention’ in care refers to ‘patients who are alive and on ART’. Patients who were transferred out are considered to be retained until the date of transfer at which time they are censored from the analysis. ‘Attrition’ is defined as discontinuation of ART for any reason, which includes died, lost to follow-up or stopped treatment.

An initial ART retention analysis was performed from the date of ART initiation. A second analysis examined the retention among patients on ART for at least 12 months in both programmes, with the start date of analysis 12 months on ART. Twelve months was chosen as cut-off point as previous studies had shown a difference in retention between ≤ 12 months and > 12 months on ART [6,7]. The end date of both analyses was set at 30 and 48 months on ART for the patients in Buhera and Thyolo respectively, to ensure a sufficient number of patients in the analysis at each time-point of treatment.

The Kaplan-Meier method was used to assess the probability of retention in ART care among the patients in both ART programmes (1) from ART initiation and (2) at least 12 months on ART, according to the initiation and follow-up site of ART (hospital or health centres). Baseline characteristics were described using medians and interquartile range (IQR) for continuous variables and counts and percentage for categorical variables. The characteristics were compared between the patient groups using the chi-square test for categorical variables and the Kruskal-Wallis test for continuous variables.

A multivariate Cox-regression model was used to estimate hazard ratios (HR) for the treatment outcomes, adjusting (aHR) for the following potential confounders: age, start year ART, gender and ART eligibility stage. All significant factors, with *P*-value <0.2, were included in this multivariate analysis for each programme.

Data analysis was done using STATA 10 (StataCorp LP, College Station, TX, USA).

### Ethics assessment

Both the Malawian and Zimbabwean ministry of health provided general oversight and approval for the collection and use of routine programme data for the monitoring and evaluation, therefore ethical approval for the type of analysis conducted in this study was not required.

## Results

In Buhera and Thyolo, respectively 9,721 and 12,004 adults were initiated on ART during the study period. In both ART programmes, respectively 74.7% and 82.5% of the patients, who had been on ART for at least 12 months, were initiated on treatment at hospital level.

### Patient characteristics

Tables 2 and 3 present the baseline characteristics of the patients on ART, at ART initiation in the district hospital and health centres in both settings. The median age was 37 years (IQR 31–45) and 35 years (IQR 29–41), in Buhera and Thyolo respectively. In both programmes, more female patients were registered in the health centres and the proportion of patients in WHO stage 4 was higher in the hospitals.

**Table 2:** **Baseline characteristics of patients on ART at ART initiation in health facilities**, **Thyolo**-**Malawi**

	**Hospital**	**Health centres**	**Total**
**Total patients on ART**	8902 (74%)	3102 (26%)	12004
**Gender**			
Male	3354 (38%)	962 (31%)	4316 (36%)
Female	5548 (62%)	2140 (69%)	7688 (64%)
**Median age **– **IQR**	34 (29–41)	35 (29–42)	35 (29–41)
**WHO stage **/ **CD4**			
WHO Stage 1–2 & CD4>200/mm^3^	193 (2%)	18 (0.6%)	211 (2%)
WHO stage 1–2 & CD4≤200/mm^3^	645 (7%)	31 (1%)	676 (6%)
WHO stage 1–2 & CD4 unknown	192 (2%)	139 (4%)	331 (3%)
Stage 3^†^	5581 (63%)	2522 (81%)	8103 (68%)
Stage 4^†^	2291 (26%)	392 (13%)	2683 (22%)
**Start year of ART**			
2003	391 (100%)	NA	391
2004	1058 (100%)	NA	1058
2005	1439 (100%)	NA	1439
2006	2281 (83%)	451 (17%)	2732
2007	2225 (62%)	1347 (38%)	3572
2008	1508 (54%)	1304 (46%)	2812

^† ^Irrespective of CD4 count.

^* ^CD4 counts were not reported separately as 68% of patients followed up in the health centres did not have CD4 counts. These are no longer done on WHO stage 3 and 4 patients, according to the Malawian guidelines.

**Table 3:** **Baseline characteristics of patients on ART at ART initiation in health facilities**, **Buhera**-**Zimbabwe**

	**Hospital**	**Hospital –Health centres**^**C**^	**Health centres**	**Total**
**Total patients on ART**	3594 (37%)	1536 (16%)	4591 (47%)	9721
**Gender**				
Male	1296 (36%)	444 (29%)	1512 (33%)	3252 (34%)
Female	2298 (64%)	1093 (71%)	3077 (67.1%)	6467 (67%)
**Median age IQR**	37 (31–44)	39 (32–47)	37 (31–45)	37 (31–45)
**WHO stage **/ **CD4**				
WHO Stage 1–2 & CD4>200/mm^3^	203 (6%)	115 (8%)	357 (8%)	675 (7%)
WHO stage 1–2 & CD4≤200/mm^3^	309 (9%)	171 (11%)	510 (11%)	990 (10%)
WHO stage 1–2 & CD4 unknown	145 (4%)	61 (4%)	402 (9%)	608 (6%)
Stage 3^†^	1792 (50%)	579 (49%)	2718 (59%)	5269 (54%)
Stage 4^†^	1140 (32%)	429 (28%)	594 (13%)	2163 (23%)
**Start year of ART**				
2005	512 (100%)	NA	NA	512
2006	1133 (99%)		9 (1%)	1142
2007	1652 (67%)		784 (32%)	2436
2008	1218 (40%)		1795 (60%)	3013
2009	616 (24%)		2002 (76%)	2618

^† ^Irrespective of CD4 count.

^C ^Hospital – Health centres are the patients who initiated ART in the hospital and were later referred to the health centres.

### Retention

Table 4 shows the difference in retention rate among the patients (1) from ART initiation and (2) at least 12 months on ART according to the ART initiation and follow-up site. In both settings, the highest drop in retention rate is noticed during the first 12 months on ART.

**Table 4:** **Retention rates according to the time on ART**, **Thyolo**–**Malawi and Buhera**-**Zimbabwe**

	**6m**	**12m**	**18m**	**24m**	**30m**	**36m**	**42m**	**48m**
**Patients from ART initiation**								
**Thyolo**								
All patients	83.8%	79.7%	77.4%	75.8%	74.5%	73.3%	72.1%	70.9%
Health centres	81.7%	78.6%	76.0%	73.5%	72.8%	-	-	-
District Hospital	84.5%	80.1%	77.8%	76.4%	75.0%	73.1%	72.1%	71.0%
**Buhera**								
All patients	87.6%	84.1%	81.9%	80.1%	78.7%			
Health centres	91.3%	88.6%	86.9%	85.2%	83.9%			
Hospital – Health centre^C^	97.5%	95.8%	94.3%	93.2%	92.3%			
District Hospital	78.9%	73.5%	70.5%	68.2%	66.4%			
**Patients at least 12 months on ART**								
**Thyolo**								
All patients	-	-	97.1%	95.2%	93.5%.	92.0%	90.5%	88.9%
Health centres	-	-	96.6%	93.5%	92.6%	-	-	-
District hospital	-	-	97.1%	95.3%	93.7%	92.2%	90.6%	89.1%
**Buhera**								
All patients	-	-	97.4%	95.3%	93.6%			
Health centres	-	-	98.1%	96.2%	94.7%			
Hospital – Health centre^C^	-	-	98.5%	97.4%	96.4%			
District Hospital	-	-	95.9%	92.7%	90.4%			

^C ^Hospital – Health centres are the patients who initiated ART in the hospital and were later referred to the health centres.

**Figure 2:**
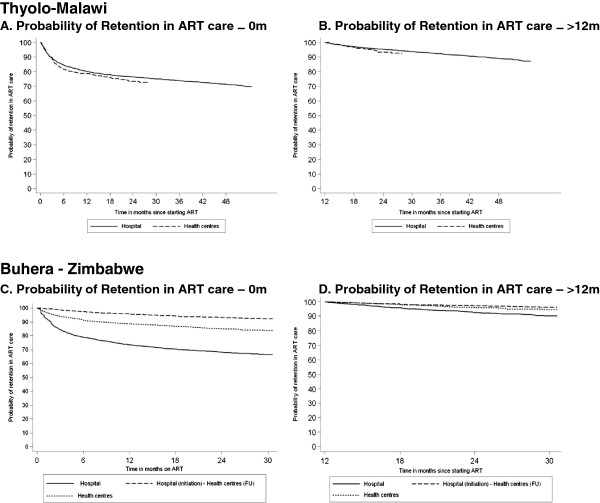
Probability of ART retention according to time on ART and ART site, Thyolo-Malawi and Buhera-Zimbabwe.

In Buhera, it was found that the patients initiated and followed up in the health centres had a 50% overall decrease in attrition hazard compared to those in the hospital (aHR 0.50, 95%CI 0.45-0.59; p-value <0.001). In Thyolo, the patients initiated on ART and followed up in the health centres had a 23% overall increase in attrition hazard, after adjustment for age, gender, ART eligibility stage and start year compared to the patients in the hospital (aHR 1.23, 95%CI 1.12-1.36, p-value <0.001). (Figure 2A-C).

Considering only the patients who had been on ART for at least 12 months, the attrition hazard among the patients initiated in the health centres (aHR 0.58, 95%CI 0.47-0.72, p-value<0.001) and those referred to the health centres (aHR 0.34, 95%CI 0.24-0.47, p-value <0.001) was found to be significantly lower than the hazard of the patients in the hospital in Buhera. The patients initiated on ART in the hospital and later referred to the health centres had a lower attrition hazard compared to those initiated on ART in the health centres (aHR 1.77, 95%CI 1.17-2.69, p-value 0.007). In Thyolo, the attrition hazard was higher in the health centres than in the hospital after adjusting for age, gender, ART eligibility and start of ART year (aHR 1.59, 95%CI 1.25-2.09, p-value <0.001) (Figure 2B-D).

### Lost to follow up and mortality

In Thyolo, the proportion of deaths among the patients on ART in the health centres decreased from 13.5% (2006) to 6.2% (2008), while the proportion of patients lost to follow up increased from 0.4% (2006) to 3.6% (2008), with the growing cohort. Among the patients initiated and followed up on ART in the hospital, both the proportion of deaths (6.5% and 2.1%%) and lost to follow up (4.0% and 2.7%) decreased respectively between 2006 and 2008.

In health centres in Buhera, the proportion of patients deceased or lost to follow up increased with the growing cohort respectively from 0.3% (2006) to 2.8% (2009) and from 1.1% (2006) to 2.4% (2009). Among patients in the hospital, the proportion of deaths (14.7% and 1.7% respectively) and lost to follow ups (6.7% and 5.6%) both dropped between 2006 and 2009.

**Figure 3:**
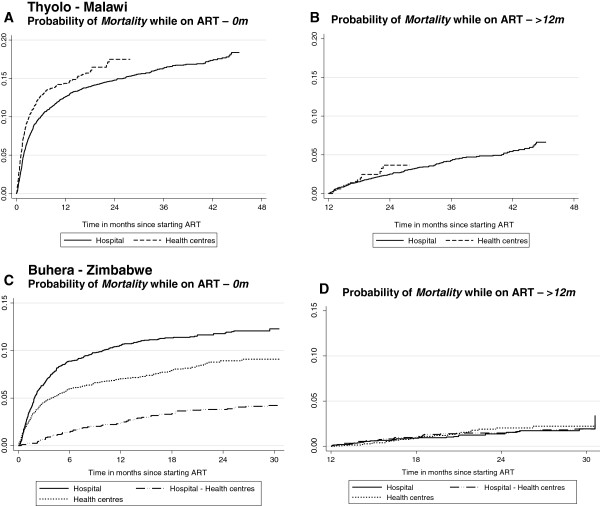
Probability of mortality while on ART according to time on ART and ART site, Thyolo-Malawi and Buhera-Zimbabwe.

**Figure 4:**
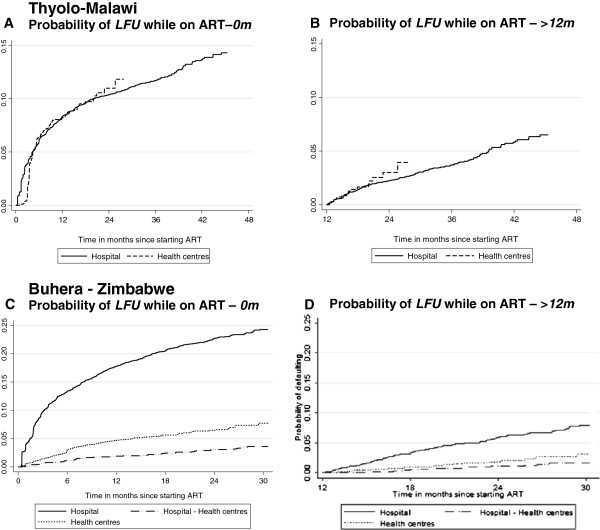
Probability of lost to follow up while on ART according to time on ART and ART site, Thyolo-Malawi and Buhera-Zimbabwe.

During the initial 12 months on ART, mortality was the main contributor to attrition in both the hospital and health centres in Thyolo. Hence, the probability of mortality was found to be significantly higher among the patients in the health centres (aHR 1.66, 95%CI 1.33-2.09, p-value <0.001). No significant difference in ‘lost to follow-up’ was found among the patients in the health centres and hospital (aHR 0.81, 95%CI 0.59-1.12, p-value 0.209). While, in Buhera, the high defaulter rate was found to be the main contributor to attrition. Patients initiated on ART in the health centres (aHR 0.25, 95%CI 0.18-0.34, p-value <0.001) or referred to the health centres (aHR 0.14, 95%CI 0.08-0.26, p-value <0.001), were found to have a decreased defaulter hazard compared to those in the hospital. The probability of mortality was lower for patients in the health centres than for the patients in the hospital (HR 0.70, 95%CI 0.56-0.86, p-value <0.001). The patients referred to the health centres were found to have a lower hazard of dying compared to those initiated on ART in the health centres (HR 0.47, 95%CI 0.36-0.62, p-value <0.001) (Figures 3A-C and 4A-C).

Considering only the patients who had been on ART for at least 12 months, no significant difference in the probability of lost to follow-up was found between the patients in the health centres and hospital in Thyolo (HR 1.25, 95%CI 0.73-2.13, p-value 0.413). While in Buhera, a significant difference in the probability of lost to follow-up was found among the patients initiated in health centres (aHR 0.42, 95%CI 0.31-0.58, p-value <0.001) and those referred to the health centres (aHR 0.23, 95%CI 0.13-0.42, p-value <0.001) compared to the patients in the hospital. In both programmes, no significant difference in risk of mortality was found according to the ART site after 12 months on ART (Thyolo - HR 1.23, 95%CI 0.74-2.05, p-value 0.416 and Buhera - HR 0.91, 95%CI 0.61-1.35, p-value 0.641) (Figures 3B-D and 4B-D).

In Buhera, the high proportion of patients on ART lost to follow up and the uncertainty of their outcomes might result in an underestimation of the mortality of all patients started on ART in the hospital or health centres. We used a web-based calculator using the meta-method to correct estimates of mortality at 1 year for lost to follow up [14]. Among all patients on ART in the hospital an uncorrected estimated mortality at 1 year of 14.9% (95% CI 12.6-17.2) and 1.7% (95% CI 1.2-2.2) was reported in 2006 and 2009 respectively. After correction, the estimates of mortality increased to 17.7% (95% CI 14.8-20.8) and 4.9% (95 CI 3.1-6.6) respectively. In the health centres, the corrected estimated mortality in 2009 increased from 1.7% (95 CI 2.4-3.2) to 4.2% (95CI 3.3-5) after adjusting for lost to follow up.

## Discussion

This analysis documents a major difference in retention rates between patients during the first 12 months on ART and patients who had been on ART for at least 12 months. In both programmes, the largest drop in retention in ART care was found during the initial 12 months on ART. After 12 months on ART, the retention rates flattened out in both settings.

### Retention outcomes compared to other programmes

The results found in this analysis are similar to those reported in previous studies. A meta-analysis of published programmes in 13 Sub-Saharan countries reported an average retention rate of 80.2% (12m), 76.1% (24m) and 72.3% (36m) [6]. Other studies conducted in Lesotho, Malawi and 11 countries in West Africa revealed similar retention outcomes: respectively 74%, 80% and 76% at 12 months and 66%, 77% and 60% at 24 months [15-17]. The national ART programme in Botswana reported survival estimates of 79.3% at 3 years on ART [18].

The main drop in retention on ART is noticed during the initial months on ART. After 12 months, the retention rate levels out over time on ART [6,7]. During the initial 12 months on ART, the contribution of mortality to the attrition rate in Thyolo amounts to 65%. In Buhera, however, 57% of the early attrition is related to the high loss to follow-up rate, which might be related to the rapid decentralisation, the lack of trained human resources available in the health centres, and the poor registration, which undoubtedly overlooked many self-referrals of patients to the health centres as soon as the ART services became available. After 12 months on ART, loss to follow-up and mortality contribute equally to the total attrition rates in Thyolo. In Buhera loss to follow-up remains the major cause of attrition (70% at 30m). The high proportion of lost to follow-up might lead to an underestimation of the mortality as well as the retention on ART. Studies in South Africa, Malawi and Zambia tracing defaulters found that 40-60% of them died within the first three months after defaulting [19-23], while other studies report that 20-80% of the traceable patients lost to follow-up were still on ART in other health facilities [24,25]. After taking into account the estimated mortality among the patients lost to follow up, the corrected mortality increased by 2.8% and 2.5% in the hospital of Buhera in 2006 and 2009, respectively.

### Treatment outcomes according to ART sites

In Buhera, the retention rate (≤12m & >12m) was significantly higher among the patients initiated on ART in the health centres and among those referred to the health centres for follow-up on ART compared to the patients in the hospital (Figure 2C-D). In the latter group, the retention outcomes were even better, although a selection bias has to be taken into account as these patients were most often stabilised in the hospital before referral. These improved outcomes in the health centres might be related to the increased proximity and accessibility of the ART care delivery services. Obvious socio-economic benefits are: faster enrolment in care, lower transport cost and time-saving [26-28]. In Thyolo, the outcomes during the initial 12 months on ART were significantly better among the patients followed up in the hospital, which is mainly due to the high early mortality in the health centres (Figure 2A-B). The more experienced health staff, more specialised care and better availability of essential drugs at the hospital level could explain this. However, after 12 months on ART no significant difference in mortality was found between the two settings. A study performed in Thyolo in 2007 found similar results and reported advanced AIDS-related complications as the main causes of death in the health centres [29]. This reinforces the importance of adequate training in recognition, early initiation of ART and prompt referral of opportunistic infections, for the staff involved in ART care delivery in the health centres [30].

### Lessons learnt

#### Similar treatment outcomes: health centre and hospital level

The treatment outcomes of both ART programmes confirm the feasibility to provide ART care with no inferior outcomes in health centres, using lower health cadres and standardised treatment schemes [15,31-35]. This rationalisation of ART care delivery allowed a significant increase in patients on ART in both settings, as ART care became more accessible.

#### Difference between short and long term retention in ART care

These findings demonstrate the importance of separating short from long term retention in care (>12m) as they are influenced by different factors. This separate analysis facilitates the identification of the major attrition causes depending on the time on ART and the adaptation of ART care delivery services accordingly. During the first months on ART more medical follow-up is required, especially for symptomatic patients. Once stable on ART, however, medical care is only needed sporadically. The main focus should then lie on regular drug refill and psychosocial support to enable patients to stay in life-long treatment [36-38]. Adapted and innovative ART care delivery models, dissociating medical follow-up from drug refill activities, can be introduced with medical care mainly offered on the demand of patients. The drug refill could be spread out more over time, every three or six months instead of monthly, and/or organised in- or outside the health structures (e.g. schools, work places community centres, etc.) depending on the context and the resources available [39-41]. Such innovative models may help to retain patients on treatment and reduce the workload of the limited number of health staff available in the health facilities, freeing up time for more severe cases [15]. However these simplified, public-health orientated models require that health staff is sufficiently trained and coached, the community is highly engaged in the ART delivery care, patients are empowered, ARV drugs supply is uninterrupted and medical care is readily available for the few cases that deteriorate [42-44]. Programmes in Mozambique, Malawi and South Africa with a strong community involvement, offering adherence support through community health workers or support groups, reported significantly better treatment outcomes [41,45-47].

#### Defaulter tracing

To improve the retention in ART care, an effective active tracing system should be a priority to locate patients who are late for their appointments, without delay [48,49]. With the growing cohorts, alternative strategies to improve patient follow-up and defaulter tracing using innovative methods, adapted to the context, need to be explored to improve the cost-effectiveness of the current strategies. Studies in Zambia and South Africa reported that up to 19 home visit attempts were required to generate one single return visit among those patients classified as late with a cost of over 200US$ per returned patient [50,51].

To avoid defaulting of patients, it is important to have good, regular updated records on patients’ contacts and a person in charge in each health facility to coordinate the defaulter tracing activities [15,52]. With the growing number of mobile phone users in the developing world, mobile phone technology increasingly presents a cheap and effective method to trace these patients [53]. A project in Kenya demonstrated improved adherence in patients contacted by mobile phone compared to those who were not [54]. In addition to being a good tracing system, mobile technology can also strengthen the referral systems between the different health facilities and facilitate the exchange of information, to ensure a continuum of care [55].

#### Monitoring system

The monitoring and evaluation of decentralised ART care delivery and referral services depends highly on regular and comprehensive patient follow up. The weak health system and the lack of motivated human resources often result in an underestimation of the actual treatment outcomes.

To enable monitoring of the growing caseload in the next years, a more simplified and operational monitoring system will be required, instead of the detailed quarterly cohort analysis currently performed in most countries [56,57]. However, to do so, more standardised definitions and indicators would be useful. Currently a number of terms, like retention, attrition and lost to follow-up, are defined ambiguously in the literature.

#### Limitations and strengths of the study

Several limitations of the study have to be taken into account. First, there is a lack of information on the patients lost to follow-up in the programme, as it remains unclear which proportion of patients may be self-referred without registration. Second, the data used was routinely collected and might include some recording errors. Third, the analysis period for the different patient groups is not identical which might cause a bias in the overall outcomes. After all, patients initiated on ART during a later year might benefit from more experienced health staff in ART care management or better organised services. Also, as the programme is longer in place, patients might present themselves at less advanced clinical stages before ART initiation. Fourth, the comparison of the retention outcomes between the patients according to the initiation and follow-up site might be biased by the selection of patients in each health facility; severely ill patients might be followed in the hospital and only be referred to the health centres when stabilised on ART. Fifth, in spite of using cohort data from two different rural districts in two different countries we feel confident about our interpretation of the difference found between early attrition and late attrition. However, a different approach and methodology would be required to explore the lessons learnt from a comparative case study analysis. The introduction of a complex intervention, such as decentralisation of care may have quite different implications and lead to different outcomes in two different countries. In fact in Malawi the decentralisation of ART to health centres was carefully planned and rolled out progressively, while in Zimbabwe it was done rapidly reacting to serious social-economic challenges. In this paper we did not focus on the comparison between the two programmes. Anyhow, we do not think our generic insights (for example on different retention rates) need substantial qualification because of the different programmes.

The strengths of this study are the large number of patients who had been on ART for at least 12 months in the study and the availability of retention outcomes at 36 and 48 months on ART, as very few studies report on long term outcomes beyond 24 or 36 months. In addition, the separate presentation of the short and long term retention rates for the different health facilities is an innovative way to better identify the causes of attrition according to the time on ART and the ART site.

## Conclusions

In conclusion, this analysis highlights a difference in retention rate of patients ≤ and > 12 months on ART, with a high attrition rate in the initial 12 months on ART, mainly related to medical problems. After 12 months on ART, the retention rate flattens, with loss to follow-up being the main cause of attrition. This suggests the need for a different approach to ART care delivery, based on the duration on ART. More innovative ART care delivery models will be required in the future to enable coping with the growing caseloads of patients on lifelong ART, to allow for further ART scale-up and improve retention in care, by involving the community and introducing drug delivery points outside the health facilities, depending on the local context. Moreover, programmes require improved systems to prevent patients from defaulting or to trace patients with a missed appointment as soon as possible, in close collaboration with the community, in order to retain patients in ART care.

## Abbreviations

aHR: Adjusted hazard ratio; ART: Antiretroviral treatment; CI: Confidence interval; HIV: Human Immunodeficiency Virus; HR: Hazard ratio; FUCHIA: Follow up and care of HIV infection and AIDS; MSF: Médecins Sans Frontières; PLWHA: People living With HIV/AIDS; SSA: Sub-Saharan Africa.

## Competing interests

We declare that we have no conflict of interest.

## Authors’ contributions

FR conceptualised the study and wrote a first draft, which was edited by all authors. MM assisted with country specific data collection. WVD checked scientific soundness and reviewed the manuscript several times. All authors – FR, WVD, MM, RZ, LL and OK contributed to the intellectual content of this article. FR finalised the manuscript. All authors read and approved the final version prior to publication.

## Funding

Both programmes in Thyolo and Zimbabwe are supported by Médecins sans Frontières.

## Pre-publication history

The pre-publication history for this paper can be accessed here:

http://www.biomedcentral.com/1472-6963/12/444/prepub
